# Correlation between the Quality of Attention and Cognitive Competence with Motor Action in Stroke Patients

**DOI:** 10.1155/2015/823136

**Published:** 2015-04-19

**Authors:** S. Arsic, Lj. Konstantinovic, F. Eminovic, D. Pavlovic, M. B. Popovic, V. Arsic

**Affiliations:** ^1^High Medical School of Professional Studies, Lole Ribara 1/2, 35230 Cuprija, Serbia; ^2^Faculty of Medicine, University of Belgrade, Doktora Subotica 8, 11000 Belgrade, Serbia; ^3^Faculty of Special Education and Rehabilitation, University of Belgrade, Visokog Stevana 2, 11000 Belgrade, Serbia; ^4^Faculty of Electrical Engineering, University of Belgrade, Bulevar Kralja Aleksandra 73, 11120 Belgrade, Serbia; ^5^Department of Physical Medicine and Rehabilitation, General Hospital, Karadjordjeva 4, 35000 Jagodina, Serbia

## Abstract

It is considered that cognitive function and attention could affect walking, motion control, and proper conduct during the walk. To determine whether there is a difference in the quality of attention and cognitive ability in stroke patients and patients without neurological damage of similar age and education and to determine whether the connection of attention and cognition affects motor skills, the sample consisted of 50 stroke patients tested with hemiparesis, involved in the process of rehabilitation, and 50 persons, randomly chosen, without neurological damage. The survey used the following tests: Trail Making (TMT A B) test for assessing the flexibility of attention; Mini-Mental State Examination (MMSE) for cognitive status; Functional Ambulation Category (FAC) test to assess the functional status and parameters of walk: speed, frequency, and length of stride; STEP test for assessing the precision of movement and balance. With stroke patients, relationship between age and performance on the MMSE test was marginally significant. The ratio of performance to TMT A B test and years does not indicate statistical significance, while statistical significance between the MMSE test performance and education exists. In stroke patients, performance on MMSE test is correlated with the frequency and length of stride walk. The quality of cognitive function and attention is associated with motor skills but differs in stroke patients and people without neurological damage of similar age. The significance of this correlation can supplement research in neurorehabilitation, improve the quality of medical rehabilitation, and contribute to efficient recovery of these patients.

## 1. Introduction

As a widespread problem, a stroke is undergoing various stages of great interest for all professionals who deal with this matter for years. According to the World Health Organization (WHO) [1], beside the diagnostic and therapeutic options, a stroke is the third most common cause of death as a disease (after cardiovascular and malignant diseases) and the second most common cause of functional disability that ranges between light (35,8%), medium (33,3%), and severe (30,9%) [2]. The quality of recovery of stroke patients in the process of rehabilitation depends on the severity of illness, appropriate therapy, and the level of preservation of cognitive and motor functions. Cognitive deficits within the stroke have multiple meanings. It is assumed that there is a conditional quality of motor function, movement, stability, balance, and walking parameters, with the degree of preservation of cognitive functions. Up until recently, it has been considered that running is mostly automatic motor task, which requires a minimum of cognitive engagement on a higher level. However, growing evidence is linking changes in attention and general cognitive engagement with the present irregularities of walk in stroke [3].

Attention can be viewed as a specific example of executive function [3]. The same author classifies the attention as* sustained attention*, which refers to the ability to sustain attention on a specific task over a period of time;* selective attention*, enabling reception of essential and elimination of irrelevant information;* divided attention*, which refers to the ability to perform more than one task at the same time; and* variable attention*, which refers to the rapid change of focus from one task to another. In the study of the same author [3], the focus was on the divided attention, and they came to the conclusion that this kind of attention plays an important role in walking during commanding multiple tasks and changing situations, while the same authors argue that there are clinical implications for the risk of falling.

The cognitive functions include perception, memory, and thinking [4]. The complexity of cognitive functions and their relative independence can be demonstrated by comparing the various dysfunctions [5].

A walk of a healthy man is a rhythmic cycle that repeats itself in a coordinated manner. The control of walking involves a large number of components of the motor system, primarily a motor cortex, basal ganglia, cerebellum, and spinal cord. In normal circumstances, a walk represents an automatic activity, in which the sequence of execution pays no attention. In cases of the central nervous system damage, as in after a stroke, these schemes or the majority of them are no longer present. Then the patient adjusts the schemes that are available and kinematic and kinetic aspects of locomotion vary depending on the severity of damage, the degree of recovery, and the use of compensatory mechanisms. The quality of walk recovery is made hierarchically arranged [6, 7]. In this study, the correlation between the quality of attention and cognitive and motor skills in patients after stroke has been examined.

## 2. Methods

### 2.1. Participants

The study was conducted at the Department of Extended Care and Treatment with Rehabilitation of the General Hospital of Cuprija and in Gerontology Center in Jagodina during the period from August 1, 2012, to March 1, 2013. The Ethics Committee of the General Hospital of Cuprija gave the approval for the research which was conducted in accordance with the ethical standards of the Helsinki Declaration. All subjects were informed about the objectives of the study and gave their consent.

We included in our group stroke patients, in whom the early rehabilitation was conducted and who upon entering the department continued with further rehabilitation measures and procedures. The study included 100 respondents, 50 ischaemic stroke patients in subacute phase who already underwent early rehabilitation, with age 50 to 80 years with hemiparesis, and 50 age and gender matched persons without neurological damage. Patients already underwent early rehabilitation. Basic methodological principle of the research is based on the comparison of results between the two groups consisting of stroke patients and persons without neurological damage (Parkinson's disease, multiple sclerosis, dementia, and depression). The aim was to examine the relationship between the quality of attention, cognitive functioning, and motor skills.

### 2.2. Cognition Assessment

Mini-Mental State Examination (MMSE) is a screening test for assessing the cognitive state of patients and is simple to use, sensitive, and valid. From the inclusion into clinical practice it has been proven as reliable and suitable for the initial assessment of mental status follow-up. MMSE examines the temporal and spatial orientation, memory skills (immediate and delayed), attention, oral and written language, and constructional abilities in two dimensions. The implementation itself lasts 10–30 minutes. The test has eleven tasks where each one scores a number of points, total score is 30 points, and the scale ranges from 0 to 30, so that there are levels of severe cognitive impairment (from 0 to 17 points); medium impairment (from 18 to 23 points); and without impairment (from 24 to 30 points). It must be taken into account that the test provides just a rough evaluation of cognitive impairment. The level of education of examinees must also be taken into account. Classifying of patients into categories of cognitive impairments according to MMSE test, we took into account the existence of the following specific standards depending on the level of education: 21 points or less indicate cognitive impairment for person with primary education, while 24 points or less indicate cognitive impairment for persons with higher education.

### 2.3. Assessment of Attention

*Trail Making Test (TMT A  B).* This is a test of the scope of neuropsychological battery of tests and is used for the evaluation of flexibility of attention. The attention is focused on orientation and concentration of mental activity to something specific, whereby the orientation determines selectivity and duration and a focus on possibility of removing distractions. Attention was tested by TMT A  B test. It has been recognized since its inception as a technique sensitive to the effects of brain damage in general. The TMT consists of two parts. TMT A requires an individual to draw lines sequentially connecting 25 encircled numbers distributed on a sheet of paper. Task requirements are similar for TMT B except that the person must alternate between numbers and letters (1, A, 2, B, 3, C, etc.). The score on each part represents the amount of time required to complete the task.

### 2.4. Assessment of Motor Skills

#### 2.4.1. Functional Ambulation Category (FAC) Test

The test is designed for assessment of quality of movement and performance of motor tasks in patients after stroke. Functional Ambulation Category (FAC) test or functional test movement is a simple measuring instrument that includes assessment of walking ability in hemiplegic patients. FAC score is as follows: 0, not walking independently; 1, assistance of one person; 2, verbal support; 3, independent on the flat ground; 4, obstacle assistance; 5, completely independent. Applying this test, the following gait parameters can be measured: walking speed (m/min), stroke rate (number of steps per min.), and stride length (cm).

#### 2.4.2. STEP Test

The test is intended for the evaluation of performance of motor tasks while overcoming certain obstacles, including precision walking and maintaining balance. The patient after a given signal (visual or auditory) tries to overcome an obstacle crossing one leg over the obstacles and returning it back. Placed obstacles are in the form of block of height 7.5 cm, width 41 cm, and depth 30 cm. Distance of obstacles from the patient is 5 cm. Within 15 seconds, the number of attempts and the number of successfully executed movements are counted. Score is determined by the number of successful and unsuccessful attempts.

### 2.5. Statistical Analysis

In this study, we have two groups of subjects. Group 1 is group of subjects with stroke and Group 2 is group of healthy subject. Sample size of both groups is 50 (*n*_A_ = 50; *n*_B_ = 50).

Descriptive statistics are represented by mean with standard deviation (SD) for continuous parameters and by percentage for categorical parameters.

For the statistical comparisons of means (medians) continuous parameters between groups, we used the independent *t*-test for two independent groups (the Wilcoxon Mann-Whitney test for two independent groups). Chi-square test or Fisher's exact test was used for categorical parameters.

Pearson's correlation (*r*) was used to study correlation between dependent (TMT A, TMT B, and MMSE) and independent (age, education level, and gender) variables. The partial correlation was done between variables TMT A, TMT B, and MMSE and variables FAC, stride length, frequency of gait, speed of gait, number of trials, number of successful executions, and balance with controlling variables age.

The multiple linear regression was used to find relationships between dependent (TMT A, TMT B, and MMSE) and independent (age, gender, and education level) variables.

Univariate analysis of covariate (ANCOVA) was used to examine the effect of age on TMT A, TMT B, and MMSE, separately, with education level as a covariate. Additionally, ANCOVA was used to examine the effect of education level on TMT A, TMT B, and MMSE, separately, with age as a covariate. Also, comparing mean of score of TMT A, TMT B, and MMSE, separately, divided by age and education level categories, separately, was done by one-way ANOVA (parametric test) or Kruskal-Wallis (nonparametric) test. For those parameters where it was found statistically significant, multiple comparisons were obtained by Tukey's test within one-way ANOVA or Holm's test within Kruskal-Wallis test. Statistical testing of hypotheses was performed by two-sided statistical tests with the significance level 0.05. The power of a test determines if the test is useful. According to power of test, we can see if the obtained results can be trusted. The desired power of test is 80% and above [8]. The power of test and sample size is closely related. In order to increase the power, the sample size needs to be increased.

RStudio software (0.98.976) and SPSS 17.0 (Chicago, IL) were used for analysis of data.

## 3. Results

### 3.1. Participants' Demographics

The groups are matched by age, gender, and education level. The statistical significant differences between means (medians) of groups exist in all other parameters. The balance divided into three categories in both groups is also statistical different and it means that groups are different according to categories (number of subjects in groups divided by categories are statistically significantly different). The marital status divided into four categories in both groups is also statistically different and it means that groups are different according to categories. Descriptive statistics of all parameters estimated in this study with finding statistical significant differences between groups are shown in Table 1.

### 3.2. Connection between Cognition and Attention to Gender, Age, Education, and Locomotion in Stroke Patients and Control Groups

In stroke patients group, Pearson's correlation between MMSE and variables age, FG, SL, gender, and education level is statistically significant, whereas between MMSE and variables NoT, NoSE, FAC, and SG statistical significance is on borderline. The *r* between TMT A and variables gender and education level is statistically significant, whereas between MMSE and SL statistical significance is on borderline. The *r* between TMT B and variables gender and education level is statistically significant (Table 2(a)).

In control group, the *r* between MMSE and all other variables is statistically significant. The *r* between TMT A and all other variables is statistically significant except between TMT A and FAC where statistical significance is on borderline. The *r* between TMT B and variables age, NoT, NoSE, FAC, SG, and education level is statistically significant. The statistical significance between TMT B and variables FG and SL is on borderline, whereas the *r* between TMT B and gender is not statistically significant (Table 2(b)).

### 3.3. Connection between Cognition, Attention, and Motor Skills in Stroke Patients and Control Groups

In stroke patients group, correlation between MMSE and FG and MMSE and SL controlled by age is statistically significant (partial correlation = −0.313; partial correlation = 0.367). Correlation between MMSE and NoT and MMSE and NoSE controlled by age is on borderline statistical significance (partial correlation = 0.242; partial correlation = 0.259). Correlation between MMSE and FAC and MMSE and SG controlled by age is not statistically significant (partial correlation = 0.204; partial correlation = −0.228). Correlation between TMT A and SL controlled by age is on borderline statistical significance (partial correlation = −0.238), whereas all other correlations are not statistically significant. Correlations between TMT B and all other parameters controlled by age are not statistically significant (Table 3(a)).

In control group, correlation between MMSE and all other parameters controlled by age are statistically significant (range of partial correlation = 0.316–0.477). Correlation between TMT A and NoSE, TMT A and FG, TMT A and SG, and TMT A and SL controlled by age is statistically significant (range of partial correlation = 0.356–0.455), whereas correlations between TMT A and NoE and TMT A and FAC are not statistically significant. Correlation between TMT B and NoE, TMT B and NoSE, and TMT B and FG controlled by age is statistically significant (range of partial correlation = 0.304–0.559). Correlation between TMT B and FAC, TMT B and FG, and TMT B and SL controlled by age is on borderline statistical significance (range of partial correlation = 0.246–0.269) (Table 3(b)).

### 3.4. The Effect of Age, Gender, and Education on the Quality of Cognition and Attention Deficits in Stroke Patients and Control Groups

Age, education level, and gender together account for 31.43% and 55.33% of the variance on MMSE in stroke patients group and control group, respectively. Education level alone accounts for 25.66% and 32.96% of the variance on MMSE in stroke patients group and control group. Age alone accounts for 4.34% and 19.60% of the variance on MMSE in stroke patients group and control group. Age, education level, and gender together account for 15.07% and 23.18% of the variance on TMT A in stroke patients group and control group, respectively. Education level alone accounts for 9.05% and 14.82% of the variance on TMT A in stroke patients group and control group. Gender alone accounts for 5.33% and 5.65% of the variance on TMT A in stroke patients group and control group. Age, education level, and gender together account for 16.15% and 52.92% of the variance on TMT B in stroke patients group and control group, respectively. Education level alone accounts for 6.55% and 35.12% of the variance on TMT B in stroke patients group and control group. Age alone accounts for 5.87% and 17.75% of the variance on TMT B in stroke patients group and control group (Table 4).

In Table 5 are shown descriptive statistics of MMSE, TMT A, and TMT B scores divided by age categories. Statistically significant differences between scores divided by age, in Group 1, exist only in MMSE score (*P* = 0.02). The powers of statistical tests for MMSE, TMT A, and TMT B were 83.09%, 17.74%, and 18.2%, respectively. The power for MMSE exceeds the desired power of 80%. The powers of tests for TMT A and TMT B were small because these tests had no statistically significant differences (*P* = 0.58, *P* = 0.32). Statistically significant differences between scores divided by age, in Group 2, exist in MMSE, TMT A, and TMT B score (*P* = 0.00; *P* = 0.02; *P* = 0.00). The powers of statistical tests for MMSE, TMT A, and TMT B were 99.79%, 46.52%, and 99.48%, respectively. The powers for MMSE and TMT B exceed the desired power of 80%, but the power for TMT A does not. We approached the obtained results for parameter TMT A, Group 2, sensitively, with desire to conform it with bigger sample.

In Table 6, are shown descriptive statistics of MMSE, TMT A, and TMT B scores divided by education level categories. Statistically significant differences between scores divided by education level in Group 1 exist in MMSE, TMT A, and TMT B scores (*P* = 0.00; *P* = 0.001; *P* = 0.001). The powers of statistical tests for MMSE, TMT A, and TMT B were 99.79%, 83.16%, and 38.21%, respectively. The powers for MMSE and TMT A exceed the desired power of 80% [8]. The power of test for TMT B was small (38.21%), so we approached this result sensitively, with desire to confirm it on bigger sample. Statistically significant differences between scores divided by education level, in Group 2, exist in MMSE, TMT A, and TMT B score (*P* = 0.00; *P* = 0.00; *P* = 0.00). The powers of statistical tests for MMSE, TMT A, and TMT B were 99.99%, 99.99%, and 99.99%, respectively. The powers for MMSE, TMT A, and TMT B exceed the desired power of 80% [8].

## 4. Discussion

The results of this study indicate that there is a correlation between the quality of cognitive skills and attention in subjects of similar age. The results also indicate that the connection between the quality of cognitive skills and attention of subjects of similar age correlates with motor skills. These results occurred comparing the correlation of cognitive abilities and quality of care for stroke patients and people without neurological damage, in relation to age, education, and gender. The quality of cognitive skills and attention in stroke patients and control groups consisting of individuals without neurological damage, matched for age, is significantly different (Table 4). To determine the statistical significance of differences in the quality of cognitive skills and attention to groups of stroke patients and a control group of similar age, we have analyzed within-group differences with respect to age but also in relation to the educational categories of respondents. In relation to age categories within the group of patients tested after stroke no statistically significant differences between the cognitive qualities (preservation) and years were found. The level of statistical significance can be seen between the two age categories (50–59 and 70–79) (Table 5). In the control group lost between all age groups, there was a statistically significant difference in the quality of cognitive abilities (Table 5). Quality of care and years of surveyed stroke patients does not indicate statistical significance; low efficiency of attention is due to neurological damage and is not correlated with age. In the control group, there was a statistically significant difference compared to all age groups, especially between the age categories 50–59 and 80–89 (Table 5). The results obtained in this study are compatible with studies that have dealt with this topic. It is believed that the age and level of education are two variables that most affect the performance TMT B test in healthy subjects and therefore obtained a high correlation in the control group. Effects of low efficiency of care for stroke patients are a consequence of neurological damage more than the impact of years, and the results are in Table 5. The results indicate that the surveyed stroke patients usually expressed low efficiency of visual selectivity and visuomotor tracking. In relation to the level of education, in stroke patients, and statistical significance between the quality of cognitive abilities and education, there are only those patients with education <4 and education of 8–12, while in the same group relationship between quality of care and education level indicates statistical significance at educational categories of respondents with education <4 and education >12 (Table 6). In the control group, there was a statistically significant difference in the quality of cognitive function and quality of care in all educational categories (Table 6). A statistically significant difference between all educational and age categories among the respondents from control groups fits with the fact that the difference increases with age subjects, level of education, and intellectual capacity [9].

The analyses of the effects of age and education level confirmed that they had a significant impact on MMSE and TMT performance. Attention in neuropsychological terms represents a prerequisite for normal cognitive functioning and is an integral part of the implementation of organized activities. In stroke patients, the quality of attention both in the domain of tenacity and in the field of vigilance disorders is reduced, and they were very susceptible to the effects of distracters, without any possibility of selection. The results of this study showed statistically significant differences in quality of attention between the two groups. Results of this study indicate that in persons without neurological damage no cognitive dysfunctions were noticed. In interviewed stroke patients, severe cognitive dysfunctions were noticed, but there were persons without cognitive impairment. This study shows that cognitive deficits as well as reduced efficiency of attention as a frequent consequence of stroke are associated with motor skills in these patients. The results of this study also indicate that the quality of cognitive skills and attention are connected with motor skills. In stroke patients, quality of cognitive functions is correlated with the frequency and length of stride walk, while the marginal correlation exists with the performance of FAC test, STEP test, and speed walk (Table 2(a)). In the control group, the quality of cognitive skills and attention is correlated with the parameters walk, as well as with the performance of the FAC test and STEP test (Table 2(b)).

Within this study we evaluated the correlation between flexibility, attention, and precision of walking while overcoming the obstacles in the examined stroke patients. Flexibility of attention assessed by visually motor task that demanded from the respondents to simultaneously monitor two different conceptual series and switch attention from one to the other proved to be of low efficiency and manifested as a weakness in the domain of variable attention. Thus, completely healthy people, when faced with obstacles during walk, focused on them and lost accuracy. The results of this study do not fully support this fact, because tested stroke patients had a good, handed attention, but not enough efficient variable attention, so that the precision was not neglected during the execution of activities. If the walk is automatic activity and does not require attention, then simultaneous execution of additional task should not affect the pace, the accuracy of the speed, or performance of any other task. Results of this study indicate that examined stroke patients who show a problem with attention flexibility during the walk will be focused not only on barriers but also on the accuracy of the walk, which means that they cannot give priority to one of the activities in terms of increasing the capacity of attention on selected activity. Also, the results of this studies show that stroke patients who exhibit problem with the flexibility of attention during the walk will be more focused not only on the obstacles in the environment in which they move but also on the accuracy of the speed and maintenance of the balance (Table 3(a)). It is believed that the effect of placing the double task has an impact on walking. Among healthy adults, dual tasks often caused the decline in performance of another task. In the case of cognitive and motor tasks, preference is given to the realization of the cognitive task. The results of this study show that the assignment of dual tasks with present cognitive deficits, except for the walking speed and apart from the walking speed, leads to a problem of frequency of walk as well as the pace length. The research study, which shows the importance of multiple tasks during walking [10], says that some changes during the walk may occur, but if you have multiple tasks for the same or similar domain and they use the same population of neurons, they will not interfere with one another.

The studies that used as predictor variables age and time on TMT B test indicate that the age of the respondents has an impact on motor skills, especially the length of stride, especially with the double task, with years of declining quality of attention and cognitive competencies which can adversely affect the movement.

Lundin-Olsson as part of a small study, based on the results of research, came to the conclusion that elderly persons who cannot at the same time “walk and talk” fall at the end, while those patients who can perform these two actions are far less prone to falling [10]. Faulkner in a study which included 377 elderly subjects observed that the performance of dual tasks and reaction time are correlated with the risk of fall. By analyzing results, they came to the conclusion that minimizing the risk of falls in high-risk individuals such as stroke patients can be prevented by reducing demand in one of the tasks [10]. Although there are differences in applying and observing responses during the realization of the double task, the general conclusion is that for walking attention is needed and the effort they put in dual tasks generally increases as walking becomes less automatic, especially in stroke patients. Certain cognitive functions will interfere with motor skills, in terms of inhibition or incentives. Effects of communication with stroke patients, during a walk, have a positive contribution to achieving basic security, which as a result have the prevailing of the functions of autonomous walking and achieving a satisfactory level of functional independence. The results of this study are compatible with the results of one research study, which included a total of 102 stroke patients in four rehabilitation centers in Netherlands and they examined walk, walking speed, and general movement in society and came to the conclusion that walking in company is a relevant and important factor for the recovery of walking after a stroke. Walking in company had a surprisingly positive effect not only on the speed of walking but also on maintaining the balance, endurance during walking, and the use of mobility aids [11].

The results of the research show the existence of the estimated speed of walking within the group in relation to the severity of cognitive impairment; that is, the examined stroke patients had a present cognitive impairment and lower speed of movement in relation to the stroke patients without cognitive impairment, a lower frequency of walking, and shorter length pace (Table 3(a)). Results of this study were obtained by estimating multivariate relationships between two sets of variables, that is, between the indicators of efficiency of attention and general cognitive functioning and movement characteristics of stroke patients, showing the existence of correlation.

In a study from a New York's Yeshiva University, which dealt with the problem of dementia, the conclusion that the disorder is associated with walking mild cognitive impairment in 31.5% of the surveyed elderly subjects was made [12]. The results of this research, as well as the results of the study, show that there is an association between mild cognitive impairment and difficulty in walking.

## Figures and Tables

**Table 1:** Descriptive statistics of estimated parameters and results of comparisons between groups.

Parameter	Stroke patients (Group 1)	Control group (Group 2)	*P* value
	*n* = 50	*n* = 50	
	Mean ± SD or *n* (%)	Mean ± SD or *n* (%)	
Age	69.9 ± 7.71	67.18 ± 9.27	0.175
Gender			0.548
Male	26 (52%)	23 (46%)	
Female	24 (48%)	27 (53%)	
Education (in year)			0.126
<4	6 (12%)	10 (20%)	
4–8	21 (42%)	18 (36%)	
8–12	20 (40%)	13 (26%)	
>12	3 (6%)	9 (18%)	
Marital status			<0.001
Married	33 (66%)	13 (26%)	
Widower	3 (6%)	11 (22%)	
Divorced	14 (28%)	18 (36%)	
Single	0 (0%)	8 (16%)	
MMSE	22.72 ± 3.33	26.56 ± 2.62	<0.001
TMT A	113.32 ± 51.5	81.82 ± 32.1	<0.001
TMT B	262.32 ± 74.5	201.98 ± 84.7	<0.001
STEP balance			<0.001
Yes	29 (58%)	49 (98%)	
Partly	4 (8%)	1 (2%)	
No	17 (34%)	0 (0%)	
FAC	2.62 ± 1.5	4.76 ± 0.43	<0.001
Frequency of gait	58.12 ± 12.15	18.62 ± 2.74	<0.001
Speed of gait	75.46 ± 23.1	21.58 ± 5.63	<0.001
Stride length	23.28 ± 5.4	52.28 ± 8.28	<0.001
STEP number of trials	2.14 ± 0.9	5.4 ± 1.3	<0.001
STEP number of successful executions	0.92 ± 0.96	4.84 ± 1.5	<0.001

**Table:** (a)

	Age	NoT^1^	NoSE^2^	FAC	FG^3^	SG^4^	SL^5^	Gender	Education
MMSE	−0.468^*^	0.265^#^	0.278^#^	0.240^#^	−0.393^*^	−0.265^#^	0.428^*^	0.319^*^	0.639^*^
TMT A	0.195^“^	−0.030^“^	−0.138^“^	0.01^“^	0.128^“^	0.179^“^	−0.274^#^	−0.356^*^	−0.420^*^
TMT B	−0.012^“^	−0.078^“^	−0.1017^“^	0.087^“^	0.082^“^	0.173^“^	−0.169^“^	−0.290^*^	−0.281^*^

^1^Number of trials. ^2^Number of successful executions. ^3^Frequency of gait. ^4^Speed of gait. ^5^Stride length. ^*^ *P* < 0.05. ^#^Borderline statistical significant *P*: 0.05–0.1. ^“^Not statistical significant *P* > 0.05.

**Table:** (b)

	Age	NoT^1^	NoSE^2^	FAC	FG^3^	SG^4^	SL^5^	Gender	Education
MMSE	−0.638^*^	0.551^*^	0.638^*^	0.391^*^	−0.372^*^	−0.543^*^	0.312^*^	0.357^*^	0.746^*^
TMT A	0.384^*^	−0.357^*^	−0.489^*^	−0.274^#^	0.426^*^	0.516^*^	−0.392^*^	−0.391^*^	−0.499^*^
TMT B	0.669^*^	−0.670^*^	−0.721^*^	−0.337^*^	0.271^#^	0.418^*^	−0.240^#^	−0.22^“^	−0.753^*^

^1^Number of trials. ^2^Number of successful executions. ^3^Frequency of gait. ^4^Speed of gait. ^5^Stride length. ^*^Statistical significant *P* < 0.05. ^#^Borderline statistical significant *P*: 0.05–0.1. ^“^Not statistical significant *P* > 0.05.

**Table:** (a)

	NoT^1^	NoSE^2^	FAC	FG^3^	SG^4^	SL^5^
MMSE	0.242^#^	0.259^#^	0.204^“^	−0.313^*^	−0.228^“^	0.367^*^
TMT A	−0.09^“^	−0.119^“^	0.036^“^	0.08^“^	0.157^“^	−0.238^#^
TMT B	−0.08^“^	−0.109^“^	0.086^“^	0.088^“^	0.176^“^	−0.177^“^

^1^Number of trials. ^2^Number of successful executions. ^3^Frequency of gait. ^4^Speed of gait. ^5^Stride length. ^*^Statistical significant *P* < 0.05. ^#^Borderline statistical significant *P*: 0.05–0.1. ^“^Not statistical significant *P* > 0.05.

**Table:** (b)

	NoT^1^	NoSE^2^	FAC	FG^3^	SG^4^	SL^5^
MMSE	0.316^*^	0.436^*^	0.319^*^	−0.396^*^	−0.477^*^	0.355^*^
TMT A	−0.191^“^	−0.356^*^	−0.203^“^	0.418^*^	0.455^*^	−0.40^*^
TMT B	−0.492^*^	−0.559^*^	−0.246^#^	0.269^#^	0.304^*^	−0.268^#^

^1^Number of trials. ^2^Number of successful executions. ^3^Frequency of gait. ^4^Speed of gait. ^5^Stride length. ^*^Statistical significant *P* < 0.05. ^#^Borderline statistical significant *P*: 0.05–0.1. ^“^Not statistical significant *P* > 0.05.

**Table 4:** Multiple linear regressions.

		Age	Education level	Gender	Total
Stroke patients (Group 1)	MMSE	4.34%	25.66%^*^	1.43%	31.43%
	TMT A	0.69%	9.05%	5.33%	15.07%
	TMT B	5.87%	6.55%	3.73%	16.15%

Control group (Group 2)	MMSE	19.60%^*^	32.96%^*^	2.77%	55.33%
	TMT A	2.71%	14.82%^#^	5.65%^#^	23.18%
	TMT B	17.75%^*^	35.12%^*^	0.05%	52.92%

^*^ *P* < 0.05 is significant. ^#^ *P* = 0.05–0.10 is of borderline significance.

**Table 5:** Results of comparisons of MMSE, TMT A, and TMT B scores divided by age categories.

	Age (in year)	50–59	60–69	70–79	80–89	*P* ^1^	Power^2^
Group 1	*n*	7	13	28	2		
	MMSE	25 ± 2.52	24 ± 2.38	21.75 ± 3.45	20 ± 4.24	0.02	83.09
	TMT A	100 ± 32.3	108.15 ± 47.5	122.2 ± 56.5	145 ± 70.71	0.58	17.74
	TMT B	239.57 ± 66.9	280.5 ± 38.87	256.8 ± 89.5	300 ± 0	0.32	18.2

Group 2	*n*	11	18	16	5		
	MMSE	28.73 ± 2.41	27.22 ± 1.86	25.38 ± 2.16	23.2 ± 1.8	0.00	99.79
	TMT A	69.64 ± 28.97	75.94 ± 36.41	90.13 ± 26.6	103.2 ± 28.9	0.02	46.52
	TMT B	109.82 ± 70.51	193.5 ± 68.81	250.1 ± 58.3	281 ± 42.49	0.00	99.48

^1^ANOVA or Kruskal-Wallis. ^2^Power of statistical test (in percent).

**Table 6:** Results of comparisons of MMSE, TMT A, and TMT B scores divided by education level categories.

Education level (duration in year)		<4	4–8	8–12	>12	*P* ^1^	Power^2^
Group 1	*n*	6	21	20	3		
	MMSE	18.83 ± 4.49	21.7 ± 2.17	24.25 ± 2.5	27.3 ± 1.15	0.00	99.79
	TMT A	162 ± 29.77	123.38 ± 55.37	102 ± 46.2	71.7 ± 8.32	0.01	83.16
	TMT B	300 ± 0	269.3 ± 90.06	254.1 ± 66.6	193.3 ± 32.3	0.01	38.21

Group 2	*n*	10	18	13	9		
	MMSE	24 ± 2.11	25.5 ± 2.15	27.9 ± 1.3	29.6 ± 1.01	0.00	99.99
	TMT A	105.9 ± 41.9	246.9 ± 25.9	63.3 ± 24.4	64.3 ± 15.1	0.00	99.99
	TMT B	276.5 ± 45.1	246.9 ± 57.8	148.7 ± 55.7	106.2 ± 59.1	0.00	99.99

^1^ANOVA or Kruskal-Wallis. ^2^Power of statistical test (in percent).

## Limitations of the Study

Limitations of this study are reflected in the limited number of stroke patients tested, because only those who were in the process of rehabilitation were chosen. Proving the existence of correlation between the quality of attention and cognitive function with motor skills did not show whether this relationship can affect motor recovery in these patients. Still, the existence of a connection or relationship between cognitive and motor aspects of patients after a stroke in the rehabilitation process provides an opportunity of application of neuropsychological rehabilitation, which would be included along with physiotherapy, cognitive training, and stimulation of social integration. However, limitation of this study could be the direction of future research, which would complement this study and give an additional contribution.
